# Dose–response association between admission neutrophil-to-lymphocyte ratio and clinical severity in *Gloydius brevicaudus* envenomation

**DOI:** 10.3389/fimmu.2026.1799662

**Published:** 2026-03-25

**Authors:** Yu-hong Wang, Xin Du, Mei-lin Hao, Xiao-qing He, Hong-sheng Liu

**Affiliations:** Department of Emergency, the Fourth Medical Center of PLA General Hospital, Beijing, China

**Keywords:** dose–response relationship, immune dysregulation, immunothrombosis, inflammatory phenotype, innate immunity, neutrophil-to-lymphocyte ratio, snakebite envenomation

## Abstract

**Background:**

Snakebite envenomation triggers a complex systemic inflammatory response; however, the immunological features underlying clinical severity remain incompletely characterized. The NLR reflects the balance between innate immune activation and adaptive immune suppression, yet its association with clinical severity in Gloydius brevicaudus envenomation has not been systematically evaluated.

**Methods:**

We performed a retrospective cohort study of 203 patients with confirmed G. brevicaudus envenomation. Clinical severity at presentation was assessed using a simplified bedside severity score based exclusively on clinical manifestations and dichotomized as mild or moderate-to-severe. Admission NLR was evaluated as a marker of inflammatory phenotype. Multivariable logistic regression models were constructed adjusting for demographic and bite-related factors. Dose–response relationships were explored using log-transformed NLR and quartile-based analyses with trend testing.

**Results:**

Patients with moderate-to-severe envenomation exhibited significantly higher admission NLR values. After adjustment for baseline clinical characteristics, log-transformed NLR remained independently associated with inflammatory severity (adjusted odds ratio [OR] 1.98, 95% confidence interval [CI] 1.39–2.81). This association persisted after additional adjustment for infection status and leukocytosis. Quartile analyses demonstrated a clear dose–response relationship, with patients in the highest NLR quartile showing a nearly fourfold increased risk of moderate-to-severe envenomation compared with those in the lowest quartile (adjusted OR 3.94, 95% CI 1.69–9.17; P for trend < 0.001).

**Conclusion:**

Elevated admission NLR is strongly associated with inflammatory severity in G. brevicaudus envenomation and exhibits a robust dose–response relationship. These findings support NLR as a readily available marker reflecting envenomation-associated immune dysregulation and highlight the contribution of neutrophil–lymphocyte imbalance to severe inflammatory phenotypes following snakebite.

## Introduction

Snakebite envenomation remains a significant global health problem, particularly in Asia, where Gloydius brevicaudus is among the most common venomous snakes responsible for human envenomation. Clinical manifestations range from mild local tissue injury to severe systemic involvement, including coagulopathy, shock, and multiorgan dysfunction (1). Despite advances in antivenom therapy, early identification of patients at risk for severe clinical courses remains challenging, as initial presentation may underestimate the magnitude of venom-induced inflammatory and systemic responses.

From an immunological perspective, snake venom represents a potent trigger of innate immune activation. Venom components such as metalloproteinases, phospholipases A_2_, and serine proteases rapidly induce tissue injury, endothelial disruption, and the release of damage-associated molecular patterns (DAMPs), leading to robust activation of innate immune pathways (2, 3). Neutrophils are among the earliest immune cells recruited to sites of envenomation and play a central role in venom-induced inflammation through degranulation, reactive oxygen species production, and formation of neutrophil extracellular traps (NETs) (4). While these responses may contribute to local containment of venom effects, excessive or dysregulated neutrophil activation has been implicated in systemic inflammation, microvascular injury, and coagulopathic complications following snakebite (5).

In parallel, severe systemic inflammation is frequently accompanied by suppression or redistribution of adaptive immune components, particularly lymphocytes. Acute lymphopenia has been described in a variety of critical inflammatory states and reflects stress-induced apoptosis, altered lymphocyte trafficking, and neuroendocrine–immune interactions (6, 7). In the context of snakebite envenomation, this imbalance between heightened innate immune activation and relative adaptive immune suppression may contribute to a distinct inflammatory phenotype associated with more severe clinical manifestations.

The NLR integrates these opposing immune processes into a single, readily available metric. Rather than reflecting leukocytosis alone, NLR captures the balance between innate immune predominance and adaptive immune attenuation, and has emerged as a robust marker of inflammatory severity across diverse conditions, including sepsis, acute lung injury, cardiovascular disease, and thrombo-inflammatory states (8–10). Importantly, NLR has been shown to correlate with endothelial dysfunction, immunothrombosis, and adverse clinical outcomes, supporting its role as a surrogate marker of systemic immune dysregulation rather than a nonspecific indicator of infection.

Although inflammatory markers have been explored in snakebite envenomation, the immunological significance of NLR in relation to clinical severity—particularly in G. brevicaudus envenomation—remains incompletely defined. Existing snakebite severity scoring systems rely primarily on clinical manifestations and bedside assessment, without direct incorporation of immune or inflammatory parameters. Whether admission NLR reflects an underlying inflammatory phenotype associated with more severe envenomation, and whether this association follows a dose–response pattern independent of infection or leukocytosis, has not been systematically investigated.

In this study, we examined the association between admission NLR and clinical severity in a retrospective cohort of patients with G. brevicaudus envenomation. By integrating multivariable modeling and quartile-based dose–response analyses, we aimed to determine whether NLR serves as a clinically accessible marker of envenomation-associated immune imbalance and inflammatory severity at presentation.

## Methods

### Study design and patients

This single-center retrospective cohort study was conducted in the Emergency Department of the Fourth Medical Center of the Chinese People’s Liberation Army General Hospital (Beijing, China), a tertiary referral center with a dedicated snakebite management service serving Northern China.

**Figure 1:**
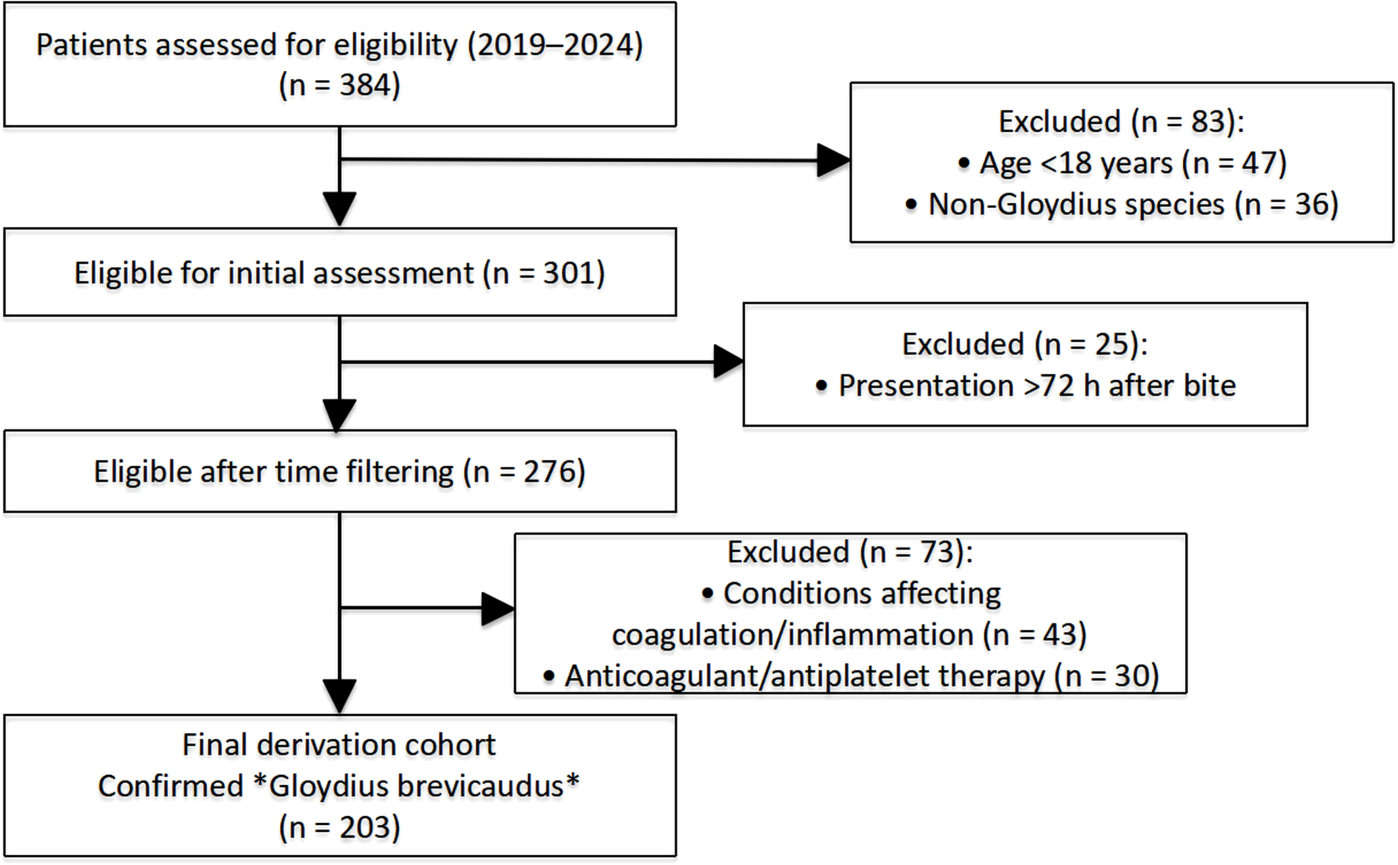
Flowchart of patient selection for the study cohort of Gloydius brevicaudus envenomation.

Electronic medical records of all patients presenting with snakebite injuries between April 1, 2019, and November 30, 2024, were systematically screened. After application of predefined inclusion and exclusion criteria, 203 patients with confirmed Gloydius brevicaudus envenomation were included in the final analytical cohort (**Figure 1**). The primary objective was to establish a well-defined cohort of patients with confirmed Gloydius brevicaudus envenomation. Inclusion criteria were: (1) age ≥18 years; (2) confirmed G. brevicaudus bite, as determined by the species identification protocol described below; and (3) availability of sufficient clinical documentation to permit assessment of snakebite severity using the simplified clinical assessment score and evaluation of venom-induced consumptive coagulopathy (VICC) based on laboratory tests obtained at hospital presentation.

Exclusion criteria included: (1) pregnancy or lactation; (2) presentation to hospital more than 72 hours after the bite; (3) pre-existing conditions likely to confound coagulation or inflammatory assessment (including active malignancy, advanced liver disease, known hematologic disorders, or chronic immunosuppression); and (4) current use of anticoagulant or antiplatelet medications.

### Snake species identification protocol

Accurate identification of the offending snake species was considered essential for the internal validity of the study. A standardized, multi-step species identification protocol was therefore implemented. When available, the dead snake was brought to the hospital by the patient or accompanying individuals, and a high-resolution digital photograph was obtained and archived in the medical record. All photographic specimens were independently reviewed by two attending physicians with formal training and experience in the identification of venomous snakes native to Northern China. Species identification was cross-validated using standardized taxonomic keys and reference images.

In cases where a physical specimen or photograph was unavailable, species attribution was based on a structured clinical and epidemiological assessment. Classification as G. brevicaudus was permitted only when both of the following criteria were satisfied: (1) the patient’s description of the snake’s morphology and behavior was consistent with G. brevicaudus; and (2) the bite occurred within the well-established geographic distribution of G. brevicaudus in Northern China, including Beijing and surrounding provinces. This approach was adopted to minimize species misclassification and reduce inadvertent inclusion of morphologically similar species, such as Gloydius intermedius or Gloydius halys. Although this strategy cannot completely exclude misclassification in a small subset of cases, it reflects routine clinical practice in regions where specimen confirmation is frequently unavailable.

Among the 203 included patients, species identification was established by photographic or physical specimen confirmation in 26 cases (12.8%), expert clinical assessment without specimen or photograph in 170 cases (83.7%), and geographic plausibility alone in 7 cases (3.5%).

### Data collection

Clinical data were extracted from electronic medical records using a standardized data collection form, with all personal identifiers removed to ensure patient confidentiality. Collected variables included demographic characteristics (age, sex, and body mass index), injury-related information (bite-to-admission time, anatomical bite site, and wound infection status at presentation), and laboratory parameters obtained within two hours of emergency department admission.

Laboratory indices included platelet count, total white blood cell count, absolute neutrophil count, absolute lymphocyte count NLR, and C-reactive protein. NLR was calculated as the absolute neutrophil count divided by the absolute lymphocyte count from the same blood draw. NLR was considered an integrated marker reflecting the balance between innate and adaptive immune responses. Importantly, all laboratory measurements used for calculation of NLR and assessment of VICC were obtained prior to antivenom administration, as part of the initial emergency evaluation.

### Definition of VICC

VICC was defined based on objective laboratory evidence of systemic activation of coagulation with consumption of clotting factors, in accordance with World Health Organization (WHO) recommendations (11) and contemporary clinical toxicology literature. Diagnosis required fulfillment of laboratory criteria in conjunction with compatible clinical features, with exclusion of alternative causes of coagulopathy.

Laboratory criteria required the presence of at least two abnormalities, including hypofibrinogenemia: (1) prolonged coagulation times (prothrombin time ≥1.5 times the upper limit of normal and/or activated partial thromboplastin time ≥1.5 times the upper limit of normal); (2) hypofibrinogenemia (plasma fibrinogen <1.5 g/L); (3) evidence of enhanced fibrinolysis (D-dimer >5.0 μg/mL or fibrin degradation products >20 μg/mL); and (4) thrombocytopenia (platelet count <100 ×10^9^/L or a rapid decline >50% from baseline).

Clinical criteria required at least one of the following: (1) spontaneous mucocutaneous or internal bleeding (e.g., epistaxis, gingival bleeding, hematuria, gastrointestinal bleeding, or intracranial hemorrhage); or (2) evidence of microvascular thrombosis or end-organ dysfunction attributable to venom effects, such as acute kidney injury or ischemic limb changes.

Patients with recent exposure to anticoagulant or antiplatelet therapy, pre-existing severe liver disease, congenital coagulation disorders, or disseminated intravascular coagulation primarily attributable to non-venom causes (e.g., sepsis) were excluded from VICC diagnosis. VICC status was determined using coagulation tests obtained within two hours of emergency department presentation, corresponding to the same early blood sampling used for NLR calculation.

### Simplified clinical assessment for snakebite severity

Snakebite severity was assessed at hospital presentation using a simplified clinical assessment score based exclusively on clinical manifestations, in accordance with established bedside criteria (16). This assessment did not incorporate hematologic, inflammatory, or coagulation laboratory parameters.

Severity categories were defined as follows: no toxicity (0–1 points), characterized by the presence of bite marks without local or systemic manifestations; mild intoxication (2–4 points), defined by local manifestations such as pain, ecchymosis, or non-progressive swelling without systemic involvement; moderate intoxication (5–7 points), characterized by progressive local swelling accompanied by systemic symptoms (e.g., nausea, vomiting, dizziness) or clinically suspected systemic involvement; and severe intoxication (≥8 points), defined by severe systemic manifestations, including neurological dysfunction, respiratory distress, or hemodynamic instability. For analytical purposes, snakebite severity was dichotomized as mild (≤4 points) and moderate-to-severe (>4 points).In routine clinical workflow, bedside severity assessment was performed by the attending physician immediately upon presentation, based solely on clinical manifestations. Blood sampling was obtained concurrently as part of standard emergency care; however, complete blood count results required for NLR calculation typically become available approximately 30 minutes later. Consequently, severity classification was completed prior to availability of laboratory data and remains blinded to and independent of NLR values.

### Statistical analysis

Statistical analyses were performed using R software (version 4.3.1) and the Free Statistics platform (FreeStats, version 2.0). Continuous variables were summarized as mean ± standard deviation for normally distributed data or median (interquartile range [IQR]) for skewed non-normally distributed data, as appropriate. Categorical variables were expressed as counts and percentages. Between-group comparisons were conducted using the χ² test or Fisher’s exact test for categorical variables, and independent-samples t test or Mann–Whitney U tests for continuous variables, as appropriate.

The primary outcome was moderate-to-severe envenomation (vs. mild) at presentation, and the primary exposure was admission NLR. The association between NLR and clinical severity was evaluated using hierarchical logistic regression models to evaluate incremental explanatory value. Given the right-skewed distribution of NLR, natural log-transformed NLR (ln[NLR]) was used in regression analyses to enhance model linearity and stabilize variance. Multivariable logistic regression models were constructed in a hierarchical manner to calculate adjusted odds ratios (aORs) with 95% confidence intervals (CIs), adjusting *a priori* for clinically relevant covariates: age, sex, body mass index, bite site, and bite-to-admission time.

Additional sensitivity analyses were performed by further adjusting for wound infection status, leukocytosis, and VICC to assess the robustness of the findings. To contextualize the independent value of NLR, C-reactive protein (CRP) was evaluated in exploratory sensitivity analyses but excluded from primary parsimonious models to avoid overfitting and preserve model interpretability.

Dose–response relationships were examined by categorizing NLR into quartiles (Q1–Q4), with the lowest quartile serving as the reference group. Trend analyses across NLR quartiles were performed by modeling quartile rank as an ordinal variable. All analyses were conducted using a complete-case approach, as no missing data were present for variables included in the analyses. A two-sided p value <0.05 was considered statistically significant.

The study protocol was reviewed and approved by the Institutional Ethics Committee of the Fourth Medical Center of the Chinese People’s Liberation Army General Hospital (Approval No. 2024KY1052-KS001). The requirement for informed consent was waived owing to the retrospective design. All procedures were conducted in accordance with the Declaration of Helsinki. This study is reported in accordance with the Strengthening the Reporting of Observational Studies in Epidemiology (STROBE) statement for observational research.

## Results

### Study population

Between April 2019 and November 2024, a total of 384 patients presenting with snakebite injuries were identified from the emergency department records and screened for cohort construction. After sequential application of predefined inclusion and exclusion criteria, 203 patients with confirmed Gloydius brevicaudus envenomation were included in the final analytical cohort.

Specifically, 83 patients were excluded at the initial screening stage due to age younger than 18 years (n = 47) or envenomation by non-Gloydius brevicaudus species (n = 36). Among the remaining 301 patients, an additional 25 were excluded because of delayed hospital presentation beyond 72 hours after the bite. Of the 276 patients subsequently assessed, 73 were further excluded owing to pre-existing conditions or medication use that could confound inflammatory or coagulation assessment, including major comorbidities (n = 43) and current use of anticoagulant or antiplatelet drugs (n = 30).

The final study population therefore consisted of 203 patients, representing 52.9% of all initially screened snakebite cases. The patient selection process and reasons for exclusion at each stage are summarized in **Figure 1**.

### Baseline characteristics

A total of 203 patients with confirmed Gloydius brevicaudus envenomation were included in the analysis (**Table 1**). According to the simplified clinical assessment, 125 patients (61.6%) were classified as having mild envenomation, while 78 patients (38.4%) were classified as having moderate-to-severe envenomation (**Table 1**). Baseline demographic and injury-related characteristics were comparable between the two groups. There were no significant differences in age (45.8 ± 15.1 vs. 45.9 ± 13.2 years, *P = 0.963*), sex distribution (*P = 0.741*), body mass index (23.9 ± 3.8 vs. 24.1 ± 3.5 kg/m², *P = 0.774*), anatomical bite site (*P = 0.353*), or bite-to-admission time (*P = 0.227*).

**Table 1:** Baseline characteristics of patients with Gloydius brevicaudus envenomation stratified by the simplified clinical assessment status.

Variables	Total	Mild	Moderate-Severe	*P*
	(n = 203)	(n = 125)	(n = 78)	
Age(years), Mean ± SD	45.8 ± 14.4	45.8 ± 15.1	45.9 ± 13.2	0.963
Gender, n (%)				0.741
Female	81 (39.9)	51 (40.8)	30 (38.5)	
Male	122 (60.1)	74 (59.2)	48 (61.5)	
body mass index(kg/m²)	24.0 ± 3.7	23.9 ± 3.8	24.1 ± 3.5	0.774
Bite Site				0.353
Upper	114 (56.2)	67 (53.6)	47 (60.3)	
Lower	89 (43.8)	58 (46.4)	31 (39.7)	
Bite Time				0.227
≤ 24h	155 (76.4)	99 (79.2)	56 (71.8)	
> 24h	48 (23.6)	26 (20.8)	22 (28.2)	
Wound status at presentation				< 0.001
Wound only	99 (48.8)	74 (59.2)	25 (32.1)	
Wound with infection	104 (51.2)	51 (40.8)	53 (67.9)	
White blood cell count				< 0.001
< 10×10^9^/L	73 (36.0)	56 (44.8)	17 (21.8)	
≥ 10×10^9^/L	130 (64.0)	69 (55.2)	61 (78.2)	
NLR, Median (IQR)	7.1 (3.4–15.2)	5.5 (3.0–10.3)	11.0 (4.6–18.9)	< 0.001
CRP (mg/L), Median (IQR)	4.0 (1.0–7.0)	3.0 (1.0–6.0)	4.2 (2.8–9.9)	0.011
VICC, n (%)				< 0.001
Without VICC	145 (71.4)	105 (84)	40 (51.3)	
With VICC	58 (28.6)	20 (16)	38 (48.7)	
Platelet count				< 0.001
< 100×10^9^/L	50 (24.6)	7 (5.6)	43 (55.1)	
≥ 100×10^9^/L	153 (75.4)	118 (94.4)	35 (44.9)	

NLR, neutrophil-to-lymphocyte ratio; VICC, venom-induced consumptive coagulopathy.

Laboratory parameters were obtained at hospital admission.

Skewed variables (NLR and CRP) are presented as median (interquartile range) and compared using the Mann–Whitney U test.

In contrast, markers reflecting inflammatory burden and systemic involvement differed substantially between severity groups. Patients with moderate-to-severe envenomation were significantly more likely to present with wound infection at admission compared with those with mild envenomation (67.9% vs. 40.8%, *P < 0.001*). Consistent with this observation, leukocytosis (white blood cell count ≥10 × 10^9^/L) was more frequently observed in the moderate-to-severe group (78.2% vs. 55.2%, *P < 0.001*). Similarly, admission C-reactive protein (CRP) levels were modestly higher in patients with moderate-to-severe envenomation compared with those with mild envenomation (median [IQR]: 4.2 [2.8–9.9] vs. 3.0 [1.0–6.0] mg/L, *P = 0.011*). Regarding immune-inflammatory markers, admission NLR was markedly higher in patients with moderate-to-severe envenomation than in those with mild envenomation (median [IQR]: 11.0 [4.6–18.9] vs. 5.5 [3.0–10.3], *P < 0.001*).

Coagulation-related abnormalities were also significantly more prevalent among patients with greater clinical severity. VICC occurred in nearly half of patients in the moderate-to-severe group, compared with 16.0% in the mild group (P < 0.001). Similarly, thrombocytopenia (platelet count <100 × 10^9^/L) was observed in 55.1% of patients with moderate-to-severe envenomation, whereas only 5.6% of patients in the mild group exhibited reduced platelet counts (P < 0.001).

### Association between admission variables and moderate-to-severe envenomation

### Univariable associations between admission characteristics and clinical severity

In univariable logistic regression analyses (**Table 2**), demographic characteristics and bite-related factors were not significantly associated with moderate-to-severe envenomation. Specifically, age (OR 1.00, 95% CI 0.98–1.02; *P = 0.963*), male sex (OR 1.10, 95% CI 0.62–1.97; *P = 0.741*), body mass index (OR 1.01, 95% CI 0.94–1.09; *P = 0.773*), lower extremity bite site (OR 0.76, 95% CI 0.43–1.35; P = 0.353), and bite-to-admission time greater than 24 hours (OR 1.50, 95% CI 0.78–2.88; *P = 0.229*) showed no statistically significant associations with clinical severity.

**Table 2:** Univariable logistic regression analysis of admission variables associated with moderate-to-severe envenomation.

Variable	OR(95% CI)	*P* value
Age(years)	1 (0.98-1.02)	0.963
Male	1.1 (0.62-1.97)	0.741
Body mass index (kg/m²)	1.01 (0.94-1.09)	0.773
Bite Site-lower	0.76 (0.43-1.35)	0.353
Bite Time >24h	1.5 (0.78-2.88)	0.229
Wound with infection	3.08 (1.7-5.57)	<0.001
White blood cell count≥ 10×10^9^/L	2.91 (1.53-5.54)	0.001
CRP (Log-transformed)	1.47 (1.08–1.99)	0.013
NLR(Neutrophil-to-lymphocyte ratio) (Log-transformed)	1.90 (1.35–2.67)	<0.001
VICC(venom-induced consumptive coagulopathy)	4.99 (2.6-9.58)	<0.001
Platelet count ≥ 100×10^9^/L	0.05 (0.02-0.12)	<0.001

Continuous biomarkers (CRP and NLR) were log-transformed due to right-skewed distributions.

In contrast, admission features reflecting infection and systemic inflammation were strongly associated with increased odds of moderate-to-severe envenomation. Patients presenting with wound infection had more than a threefold higher likelihood of moderate-to-severe clinical presentation compared with those without infection (OR 3.08, 95% CI 1.70–5.57; *P < 0.001*). Consistently, leukocytosis, defined as a white blood cell count ≥10 × 10^9^/L, was also significantly associated with greater severity (OR 2.91, 95% CI 1.53–5.54; *P = 0.001*). Notably, admission log-transformed lnNLR was significantly associated with clinical severity on a continuous scale (OR 1.90, 95% CI 1.35–2.67; *P < 0.001*). Similarly, log-transformed C-reactive protein (CRP) was modestly associated with increased odds of moderate-to-severe envenomation (OR 1.47, 95% CI 1.08–1.99; *P = 0.013*).

Markers of coagulation disturbance were likewise strongly associated with severity classification. The presence of VICC was associated with a nearly fivefold increase in the odds of moderate-to-severe envenomation (OR 4.99, 95% CI 2.60–9.58; *P < 0.001*). Conversely, a platelet count ≥ 100×10^9/^L was inversely associated with moderate-to-severe classification (OR 0.05, 95% CI 0.02–0.12; *P < 0.001*). Our findings provide clinical support for the proposed immunothrombotic pathway contributing to snakebite severity.

### Multivariable association between admission NLR and moderate-to-severe envenomation

Multivariable logistic regression models were constructed to evaluate the independent association between admission NLR and moderate-to-severe envenomation (**Table 3**). Correlation analysis demonstrated only weak correlation between CRP and NLR. Variance inflation factors (VIFs) for all variables were < 3, indicating no evidence of problematic multicollinearity. All models were adjusted for age, sex, body mass index, bite site, and bite-to-admission time. In Model 1, which included log-transformed NLR in addition to baseline covariates, higher admission NLR was significantly associated with increased odds of moderate-to-severe envenomation (adjusted OR 1.98, 95% CI 1.39–2.81; *P < 0.001*). This association remained statistically significant after further adjustment for wound infection at presentation in Model 2 (adjusted OR 1.66, 95% CI 1.13–2.44; *P = 0.009*), indicating that the relationship between NLR and clinical severity was not solely explained by concomitant infection.

**Table 3:** Hierarchical multivariable logistic regression models assessing the association between admission NLR and moderate-to-severe envenomation.

Variable	Model 1 Adjusted OR (95% CI)	P value	Model 2 Adjusted OR (95% CI)	P value	Model 3 Adjusted OR (95% CI)	P value	Model 4 Adjusted OR (95% CI)	P value
NLR(log)	1.98(1.39–2.81)	<0.001	1.66 (1.13–2.44)	0.009			1.65 (1.11-2.47)	0.014
Wound infection			2.12 (1.09–4.13)	0.026	2.26(0.93–5.48)	0.070	2.07(0.84–5.10)	0.113
WBC ≥10 ×10^9^/L					1.56 (0.59–4.08)	0.369	1.04(0.37–2.92)	0.936

All models were adjusted for age, sex, body mass index, bite site, and bite-to-admission time.

Model 1: additionally included log-transformed NLR.

Model 2: additionally included wound infection at presentation.

Model 3: included wound infection and leukocytosis (WBC ≥10 ×10^9^/L), without NLR.

Model 4: included wound infection, log-transformed NLR, and leukocytosis.

Wound infection at presentation was associated with increased odds of moderate-to-severe envenomation when added to the NLR-adjusted model (Model 2: adjusted OR 2.12, 95% CI 1.09–4.13; *P = 0.026*). However, when NLR was excluded and leukocytosis (white blood cell count ≥10 × 10^9^/L) was included instead (Model 3), wound infection did not retain statistical significance (adjusted OR 2.26, 95% CI 0.93–5.48; *P = 0.070*), and leukocytosis itself was not independently associated with clinical severity (adjusted OR 1.56, 95% CI 0.59–4.08; *P = 0.369*), highlighting the limited discriminatory value of conventional inflammatory markers in the absence of NLR. In the fully adjusted model including wound infection, log-transformed NLR, and leukocytosis simultaneously (Model 4), NLR remained independently associated with moderate-to-severe envenomation (adjusted OR 1.65, 95% CI 1.11–2.47; *P = 0.014*). In contrast, neither wound infection (adjusted OR 2.07, 95% CI 0.84–5.10; *P = 0.113*) nor leukocytosis (adjusted OR 1.04, 95% CI 0.37–2.92; *P = 0.936*) showed a significant independent association.

Collectively, these findings demonstrate that admission NLR is independently associated with clinical severity in patients with Gloydius brevicaudus envenomation, and that this association persists after accounting for infection status and conventional inflammatory markers (**Table 3**). No significant interaction was observed between admission NLR and time from bite to admission (*P* for interaction = 0.964), and the direction of association was consistent across time strata (**Supplementary Figure 1**). In additional exploratory analyses incorporating log-transformed CRP (**Supplementary Table 1**), admission NLR remained independently associated with moderate-to-severe envenomation, indicating robustness of the primary findings.

### Dose–response association between admission NLR quartiles and clinical severity

**Figure 2:**
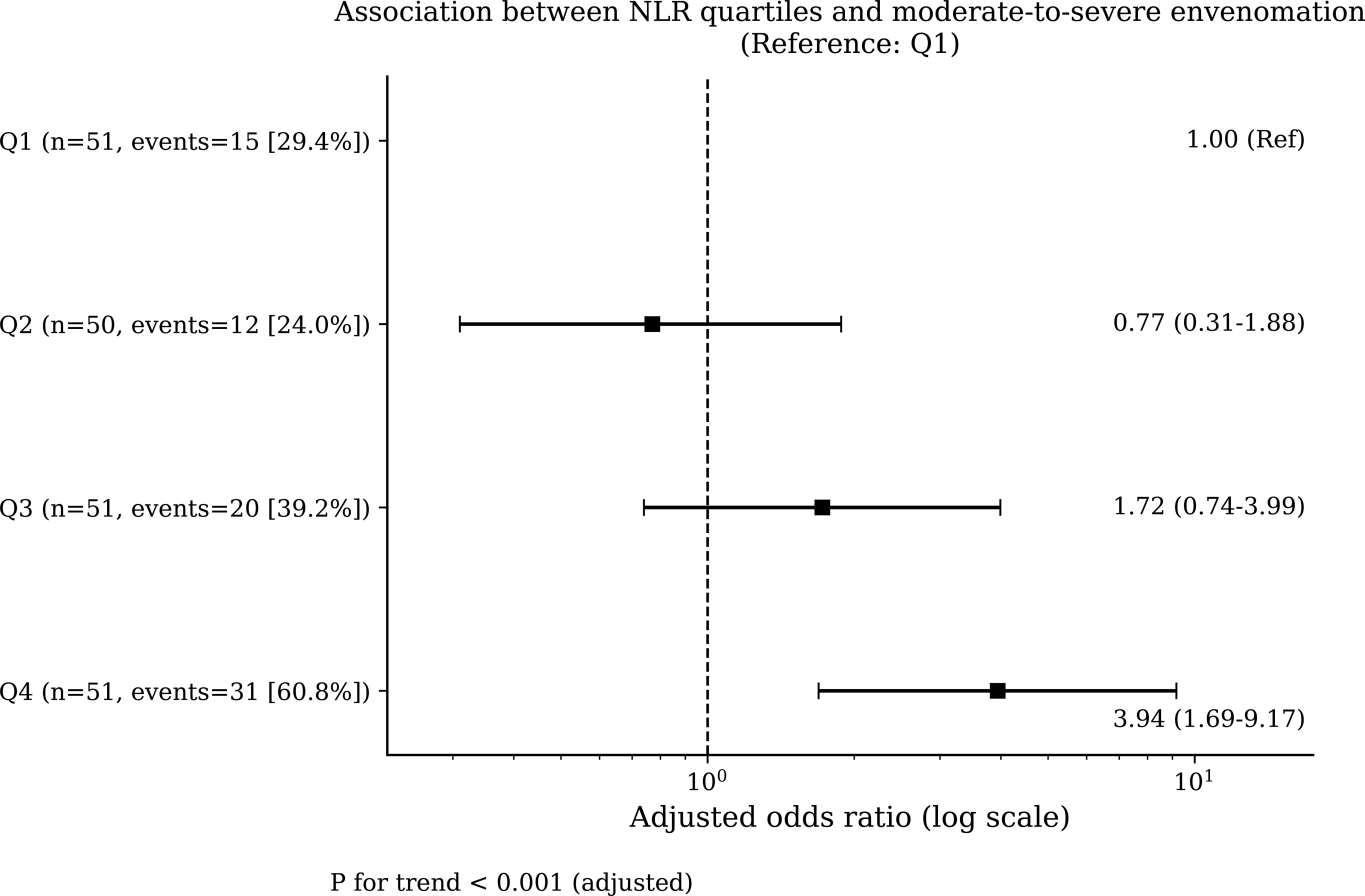
Dose–response association between admission NLR quartiles and moderate-to-severe envenomation. Adjusted odds ratios (aORs) and 95% confidence intervals were derived from multivariable logistic regression models with the lowest quartile (Q1) as the reference.

A clear dose–response relationship between admission NLR and clinical severity was demonstrated in quartile-based analyses (**Table 4**, **Figure 2**). Compared with patients in the lowest NLR quartile (Q1), those in the highest quartile (Q4) had an approximately fourfold odds risk of moderate-to-severe envenomation (adjusted OR 3.94, 95% CI 1.69–9.17). A significant linear trend across increasing NLR quartiles was observed (*P* for trend <0.001), supporting a graded association between systemic inflammatory burden and envenomation severity.

**Table 4:** Dose–response association between admission NLR quartiles and moderate-to-severe envenomation.

NLR quartile	Adjusted OR (95% CI)	*P* value
Q1(lowest)	1(reference)	
Q2	0.77 (0.31-1.88)	0.564
Q3	1.72 (0.74-3.99)	0.209
Q4(highest)	3.94 (1.69-9.17)	0.001
*P* for trend	1.65 (1.25-2.18)	<0.001

### Sensitivity analyses incorporating coagulation-related phenotypes

In sensitivity analyses accounting for coagulation-related phenotypes, the association between admission NLR and moderate-to-severe envenomation remained statistically robust. After additional adjustment for VICC, log-transformed NLR remained marginally associated with disease severity (adjusted OR 1.49, 95% CI 1.01–2.22; *P = 0.047)*, consistent with partial biological overlap between early inflammatory activation and downstream coagulopathic manifestations. Similarly, adjustment for thrombocytopenia (platelet count <100 ×10^9^/L) did not attenuate the association between NLR and severity (adjusted OR 2.13, 95% CI 1.32–3.43; *P = 0.002)* (**Supplementary Table 2**).

In exploratory analyses, admission NLR showed a modest but statistically positive correlation with the total antivenom dose administered during hospitalization (Spearman ρ= 0.207, *P = 0.003*; **Supplementary Table 3**).

## Discussion

In this retrospective cohort study of patients with Gloydius brevicaudus envenomation, we found that the NLR measured at hospital presentation was independently associated with clinical severity as assessed by a simplified bedside scoring system. This association remained robust after adjustment for demographic characteristics, bite-related factors, wound infection status, and conventional inflammatory markers, and persisted across multiple sensitivity analyses incorporating coagulation-related phenotypes. Importantly, a clear dose–response relationship was observed, with progressively higher odds of moderate-to-severe envenomation across increasing NLR quartiles. Together, these findings support a graded association between early innate immune activation and clinical severity following snakebite envenomation.

### Immunological interpretation: what does NLR represent in snakebite envenomation?

The NLR is increasingly recognized as an integrated marker reflecting the balance between innate immune activation and adaptive immune suppression, rather than a surrogate of leukocytosis alone (8, 9). In the context of snakebite envenomation, this balance may be particularly relevant, as venom exposure triggers an immediate and complex host response involving inflammatory cell recruitment, endothelial activation, and microvascular injury (1–3).

An elevated NLR at hospital presentation likely reflects early predominance of neutrophil-driven innate immune responses accompanied by relative lymphocyte depletion. Experimental and clinical studies have shown that viperid venoms, including those from Gloydius species, can directly activate neutrophils and promote the formation of NETs (4). NET release has been shown to contributes to endothelial injury, microvascular obstruction, and amplification of local and systemic inflammation, thereby linking immune activation with tissue damage and vascular dysfunction (12, 13). In parallel, acute stress responses mediated by catecholamines and glucocorticoids induce lymphocyte apoptosis and redistribution, resulting in transient lymphopenia and further elevation of NLR (6, 7). Thus, NLR may capture both arms of venom-induced immune dysregulation—excessive innate activation and relative adaptive suppression—within a single, clinically accessible parameter.

We observed a clear dose–response relationship, in which patients in the highest NLR quartile (Q4, >15.27) exhibited an approximately fourfold increase in the odds of moderate-to-severe envenomation compared with those in the lowest quartile (adjusted OR 3.94, 95% CI 1.69–9.17; P for trend < 0.001). This graded pattern further supports the role of NLR as a quantitative indicator of envenomation intensity.

**Figure 3:**
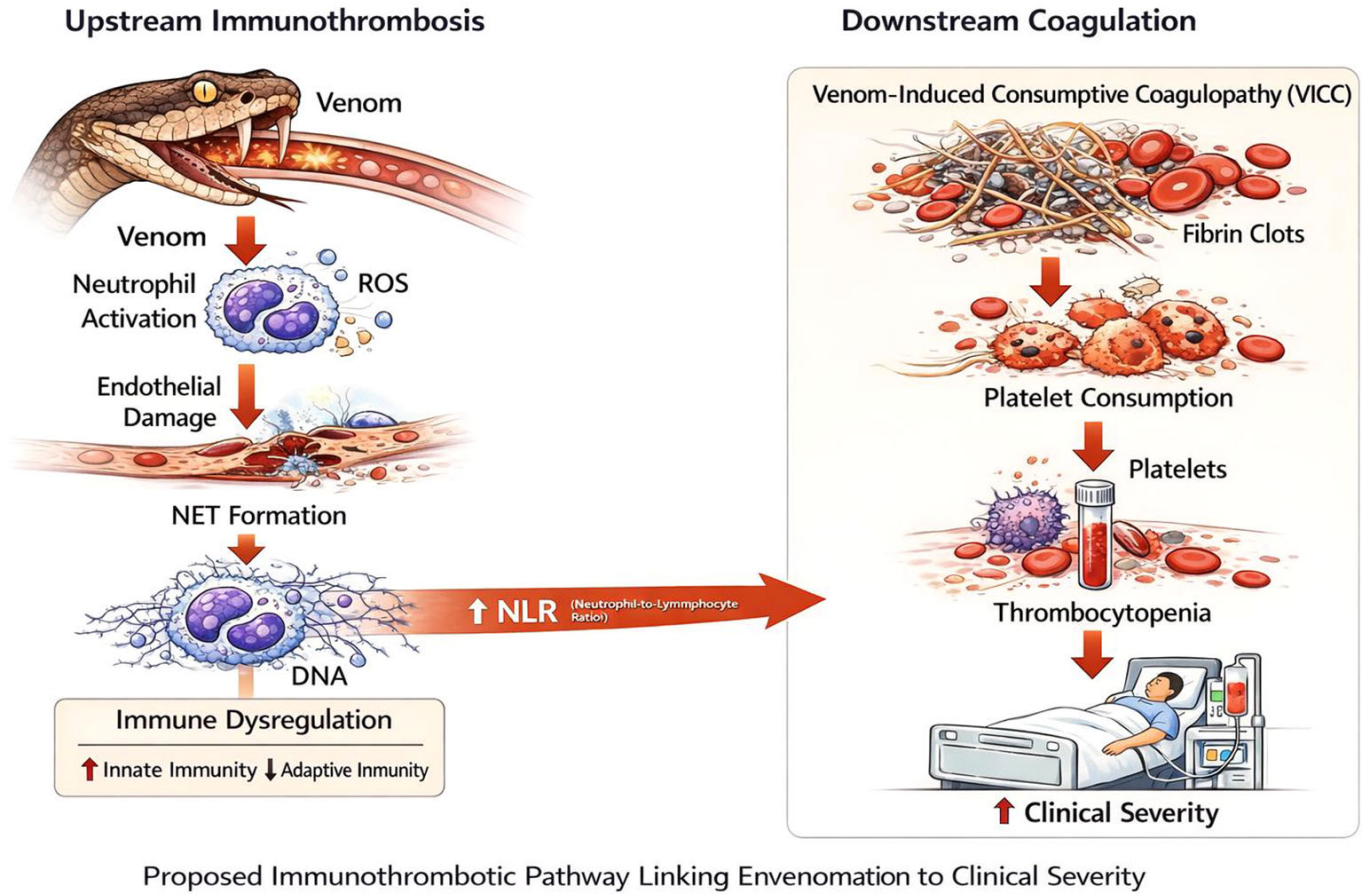
Conceptual framework linking neutrophil–lymphocyte imbalance to immunothrombosis.

Accordingly, based on our findings and existing evidence, we propose a conceptual immunothrombotic framework linking early neutrophil–lymphocyte imbalance to downstream coagulation phenotypes and clinical severity (**Figure 3**). Snake venom exposure is known to trigger early innate immune activation characterized by neutrophil recruitment, reactive oxygen species (ROS) release, endothelial injury, and formation of NETs. Concurrent stress-related lymphocyte depletion may contribute to an elevated NLR, reflecting upstream immune dysregulation. These immunothrombotic processes promote microvascular injury and activation of coagulation cascades, leading to downstream phenotypes such as VICC and platelet consumption. Collectively, this cascade provides a biologically plausible link between early immune imbalance and increased clinical severity following envenomation.

### Incremental value beyond conventional inflammatory markers

Our findings suggest that NLR provides discriminatory information beyond that of total white blood cell count alone. While leukocytosis reflects an absolute increase in circulating immune cells, it does not capture the relative contribution of different leukocyte subpopulations or the functional balance between inflammatory and regulatory immune responses (9, 10). This distinction is particularly relevant in acute envenomation, where total white blood cell counts may be influenced by non-specific factors such as dehydration, stress responses, or concomitant infection.

Consistent with this concept, leukocytosis did not retain an independent association with clinical severity in multivariable models, whereas NLR remained robustly associated after adjustment for infection status and leukocyte count. These findings support the notion that NLR reflects venom-related immune imbalance more precisely than conventional inflammatory indices and aligns with observations from sepsis, critical illness, and thrombo-inflammatory states, where NLR outperforms absolute leukocyte counts in prognostic discrimination (8–10).

Furthermore, although C-reactive protein (CRP) is a classic acute-phase reactant, our sensitivity analyses demonstrated that the association between NLR and severity remained independent after adjustment for log-transformed CRP. This finding suggests that NLR captures a distinct dimension of the host response—specifically the imbalance between innate and adaptive immunity—which may provide incremental prognostic information beyond generalized systemic inflammation reflected by CRP.

### Relationship between immune imbalance and coagulation disturbance: an immunothrombosis perspective

Coagulation abnormalities, including VICC and thrombocytopenia, are hallmark complications of G. brevicaudus envenomation and were strongly associated with clinical severity in univariable analyses. However, these manifestations likely represent downstream phenotypes arising from venom-induced endothelial injury and immune-mediated microvascular dysfunction rather than primary drivers of severity (14–17).

The attenuation, but persistence, of the NLR–severity association after adjustment for VICC or platelet depletion supports this interpretation. Neutrophil activation and NET formation are central components of immunothrombosis, promoting platelet activation, tissue factor exposure, and impairment of endogenous fibrinolysis (18–20). From this perspective, elevated NLR may reflect upstream immunothrombotic activity that precedes and contributes to overt coagulation abnormalities. Accordingly, VICC and thrombocytopenia may be viewed as downstream expressions of a shared immune–vascular injury axis rather than competing explanatory variables.

Within the proposed immunothrombotic framework, VICC is best viewed as a downstream manifestation of venom-induced coagulopathy that may lie on the causal pathway linking early inflammatory activation to clinical severity. The attenuation observed after adjustment for VICC is therefore not unexpected and may reflect partial mediation rather than pure confounding. These findings suggest that NLR captures upstream immunoinflammatory activation that contributes to, but is not fully explained by, overt consumptive coagulopathy.

Importantly, our interaction analysis showed no significant modification of the NLR–severity association by the time elapsed from bite to admission (P for interaction = 0.964). This temporal stability suggests that admission NLR may serve as a reliable biomarker for risk stratification across the early clinical window within 72 hours after envenomation. However, the observational design of the present study precludes definitive causal inference regarding these biological pathways.

### Clinical implications

From a clinical standpoint, our findings highlight the potential utility of admission NLR as a simple and readily available marker for early risk stratification in snakebite patients. Unlike specialized coagulation assays or venom-specific diagnostics, NLR can be rapidly calculated from routine complete blood counts obtained at presentation, making it particularly suitable for emergency settings and resource-limited environments (21,22).

The observed dose–response relationship across NLR quartiles further supports its clinical relevance, suggesting that NLR may aid not only in dichotomous severity classification but also in identifying gradations of risk. Integration of NLR into existing bedside assessment frameworks may enhance early recognition of patients at higher risk for severe envenomation, thereby informing decisions regarding monitoring intensity, antivenom administration, and early referral.

Beyond its association with concurrent severity, admission NLR was positively correlated with the total antivenom dose administered during hospitalization (ρ = 0.207, P = 0.003), suggesting that early immune profiling using NLR may reflect overall envenomation burden and subsequent treatment requirements.

### Strengths and limitations

This study has several strengths. First, we employed a clinically grounded severity assessment based exclusively on bedside manifestations, avoiding circularity with laboratory variables used in exposure or outcome definitions. Second, all laboratory indices, including NLR and coagulation parameters, were obtained prior to antivenom administration, minimizing treatment-related confounding. Third, the robustness of our findings was supported by multiple complementary analytical approaches, including hierarchical multivariable modeling, dose–response analyses, and sensitivity analyses incorporating coagulation-related phenotypes.

Several limitations should be acknowledged. The retrospective single-center design may limit generalizability, and residual confounding cannot be entirely excluded. Species identification relied predominantly on expert clinical assessment rather than universal specimen confirmation.Although this approach reflects real-world practice, it may have introduced a small degree of misclassification bias.

In addition, NLR was assessed at a single time point; dynamic immune trajectories during hospitalization were not captured. Future prospective studies are warranted to validate these findings and to elucidate the temporal relationship between immune activation, immunothrombosis, and clinical outcomes.

## Conclusion

In conclusion, admission NLR is independently and dose-dependently associated with clinical severity in patients with Gloydius brevicaudus envenomation. By reflecting early innate immune activation and immune imbalance, NLR provides clinically meaningful information beyond conventional inflammatory markers and coagulation indices. These findings support the role of NLR as a practical immunological biomarker for early risk stratification in snakebite envenomation and underscore the importance of host immune responses in determining clinical severity.Our findings provide clinical support for the proposed immunothrombotic pathway contributing to snakebite severity.

## Data Availability

The original contributions presented in the study are included in the article/**Supplementary Material**. Further inquiries can be directed to the corresponding author.
