# Supplementary material for: Dose–response association between admission neutrophil-to-lymphocyte ratio and clinical severity in Gloydius brevicaudus envenomation
Source: Front Immunol. 2026 Mar 25;17:1799662. doi: 10.3389/fimmu.2026.1799662 (PMC13056643; doi:10.3389/fimmu.2026.1799662)
Supplement: Supplementary file 2 [file DataSheet2.doc]

**Supplementary Materials**

**Impact Statement**

Snakebite envenomation triggers a complex systemic inflammatory response, yet clinically accessible biomarkers that reflect immune dysregulation remain limited. In this retrospective cohort of patients with Gloydius brevicaudus envenomation, we demonstrated a robust and independent dose–response association between admission neutrophil-to-lymphocyte ratio (NLR) and clinical severity. By positioning NLR as a marker of innate–adaptive immune imbalance rather than a nonspecific infection indicator, our findings provide immunological insight into the immunoinflammatory phenotype of viper envenomation. These results support the use of admission NLR as a simple and practical adjunct for early risk stratification in snakebite patients, particularly in resource-limited emergency settings.


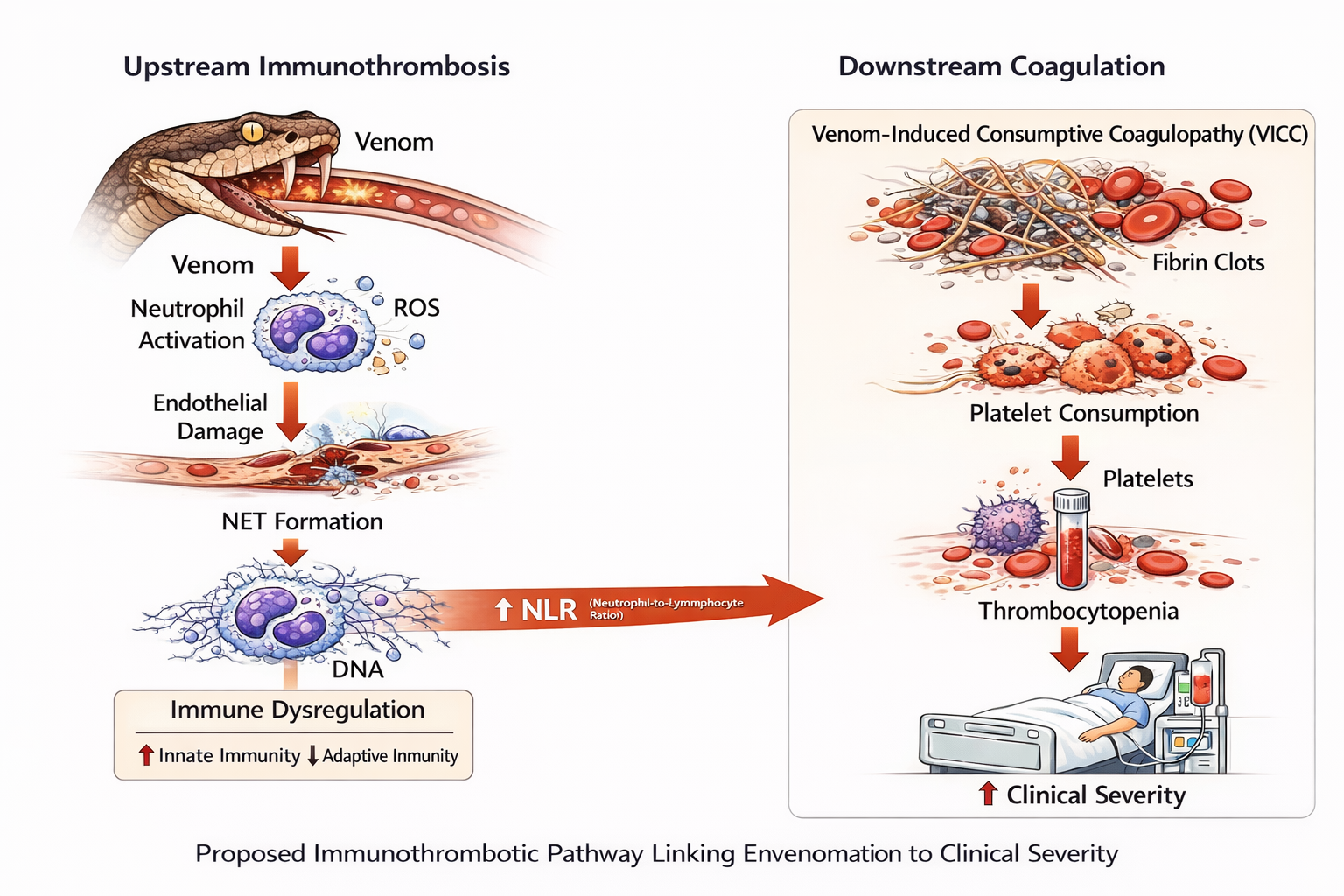


Figure3. Conceptual framework linking neutrophil–lymphocyte imbalance to immunothrombosis

Supplementary Table S1. Extended multivariable logistic regression models incorporating C-reactive protein (CRP) for the association between admission neutrophil-to-lymphocyte ratio (NLR) and moderate-to-severe envenomation

| **Variable** | **Model 1** | **Model 2** | **Model 3** | **Model 4** | **Model 5**  **(+CRP)** | **Model 6**  **(Full)** |
| --- | --- | --- | --- | --- | --- | --- |
| ln[NLR] | 1.98 (<0.001) | 1.61 (0.023) | — | 1.59 (0.029) | 1.97 (<0.001) | 1.63 (0.024) |
| ln[CRP] | — | — | — | — | 1.47 (0.021) | 1.45 (0.030) |
| Wound infection | — | 1.93 (0.075) | 2.29 (0.081) | 1.65 (0.317) | — | 1.83 (0.236) |
| WBC ≥ 10×10⁹/L | — | — | 1.51 (0.422) | 1.27 (0.648) | — | 1.03 (0.962) |

Abbreviations: NLR, neutrophil-to-lymphocyte ratio; CRP, C-reactive protein; WBC, white blood cell count.

Note: Values are presented as adjusted odds ratios (aORs) with P values in parentheses. All models were adjusted for age, sex, body mass index, bite site, and bite-to-admission time. Model 5 additionally included log-transformed CRP. Model 6 represents the fully adjusted model including NLR, CRP, wound infection, and leukocytosis.

The independent association between admission NLR and moderate-to-severe envenomation was further examined using a stepwise multivariable modeling approach (Table S1). In the primary adjusted model (Model 1), ln[NLR] was significantly associated with severity (aOR 1.98, P < 0.001). This association remained statistically significant after sequential adjustment for wound infection (Model 2, aOR 1.61, P = 0.023) and conventional inflammatory markers including leukocytosis (Model 4, aOR 1.59, P = 0.029). When C-reactive protein (CRP) was incorporated into the extended models, the magnitude of association for ln[NLR] remained largely unchanged (Model 6: aOR 1.63, P = 0.024), whereas conventional markers such as leukocytosis did not retain statistical significance.

Collectively, these findings suggest that admission NLR captures inflammatory information that is not fully explained by CRP or absolute white blood cell counts, supporting its incremental value within the multivariable framework

Supplementary Table S2. Sensitivity analyses evaluating the association between log-transformed NLR and moderate-to-severe envenomation after additional adjustment for coagulation-related phenotypes

| Variable | Additional adjustment | Adjusted OR (95% CI) | *P* value |
| --- | --- | --- | --- |
| Model 5a | VICC | 1.49(1.01-2.22) | 0.047 |
| Model 5b | Thrombocytopenia | 2.13(1.32-3.43) | 0.002 |

Abbreviations: NLR, neutrophil-to-lymphocyte ratio; VICC, venom-induced consumptive coagulopathy.

Note: Models additionally included log-transformed NLR and wound infection at presentation and were adjusted for age, sex, body mass index, bite site, and bite-to-admission time.

Sensitivity analyses incorporating coagulation-related phenotypes To further evaluate the robustness of the association between admission neutrophil-to-lymphocyte ratio (NLR) and clinical severity, sensitivity analyses were performed by additionally adjusting for coagulation-related phenotypes that were strongly associated with disease severity in univariable analyses (Table S2). In Model 5a, which included log-transformed NLR, wound infection at presentation, and venom-induced consumptive coagulopathy (VICC) in addition to baseline covariates, log-transformed NLR remained marginally associated with moderate-to-severe envenomation (adjusted OR 1.49, 95% CI 1.01–2.22; P = 0.047). This attenuation in effect size compared with the primary models suggests that part of the association between NLR and disease severity may be shared with downstream coagulation disturbances. In Model 5b, which incorporated log-transformed NLR, wound infection at presentation, and thrombocytopenia (platelet count <100 ×10⁹/L), the association between NLR and moderate-to-severe envenomation remained statistically significant and of greater magnitude (adjusted OR 2.13, 95% CI 1.32–3.43; P = 0.002). These findings indicate that the relationship between NLR and clinical severity is not fully explained by platelet depletion alone. Overall, the persistence of the NLR–severity association across sensitivity models adjusting for distinct coagulation phenotypes supports the robustness of NLR as an independent inflammatory marker associated with more severe clinical presentation in Gloydius brevicaudus envenomation.

Supplementary Table S3. Spearman correlation between admission neutrophil-to-lymphocyte ratio (NLR) and total antivenom dose during hospitalization

| **Variable** | Spearman ρ | P value |
| --- | --- | --- |
| Admission NLR and total antivenom dose | 0.207 | 0.003 |

Abbreviations: NLR, neutrophil-to-lymphocyte ratio.

Note: Correlation was assessed using Spearman rank correlation analysis. Admission NLR was calculated from the initial complete blood count obtained prior to antivenom administration. This analysis was exploratory in nature.

In exploratory analyses, admission NLR showed a modest but statistically significant positive correlation with the total antivenom dose administered during hospitalization (Spearman ρ = 0.207, P = 0.003; Supplementary Table S3).

Figure S1. Time-stratified association between admission neutrophil-to-lymphocyte ratio (NLR) and moderate-to-severe envenomation.

**
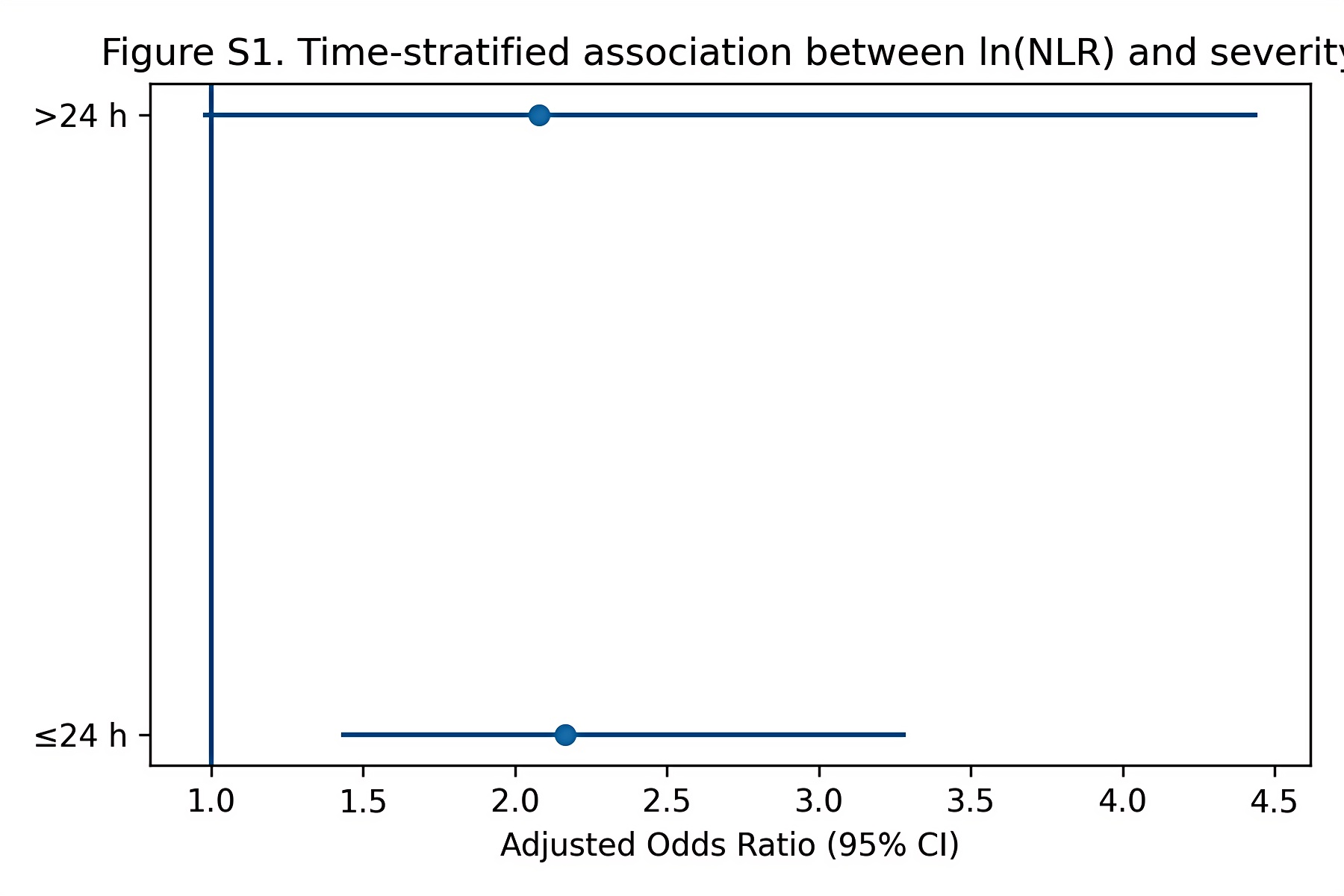
**

Forest plot showing adjusted odds ratios (aORs) and 95% confidence intervals for the association between log-transformed admission NLR and moderate-to-severe envenomation, stratified by bite-to-admission time (≤24 h vs >24 h). Estimates were derived from multivariable logistic regression models adjusted for age, sex, body mass index, bite site, and bite-to-admission time. No significant interaction between NLR and time from bite to admission was observed (P for interaction = 0.964), indicating temporal stability of the association.
